# Health-economic evaluation of an AI-powered decision support system for anemia management in in-center hemodialysis patients

**DOI:** 10.1186/s12882-025-04298-7

**Published:** 2025-08-28

**Authors:** Afschin Gandjour, Christian Apel, Dana Kendzia, Luca Neri, Francesco Bellocchio, Len Usvyat, John Larkin, Jovana Petrovic Vorkapic

**Affiliations:** 1 Frankfurt School of Finance & Management, Frankfurt, Germany; 2 Market Access, Health Economics & Political Affairs, Fresenius Medical Care Deutschland GmbH, Bad Homburg, Germany; 3 Clinical Advanced Analytics, Global Medical Office, Fresenius Medical Care Italia S.p.A., Palazzo Pignano Cremona, Italy; 4 Global Medical Office, Fresenius Medical Care, Waltham, MA USA

**Keywords:** Cost-effectiveness analysis, Economic evaluation, End-stage kidney disease (ESKD), Anemia management, Erythropoiesis stimulating agent (ESA), Artificial intelligence (AI), Personalized medicine

## Abstract

**Background:**

The Anemia Control Model (ACM) is a decision support system powered by an artificial intelligence core designed to assist nephrologists in managing anemia therapy for in-center hemodialysis (HD) patients. This study aims to evaluate the cost-effectiveness of the ACM compared to standard of care in Germany, defined as the absence of ACM and a hemoglobin (Hb) target achievement rate of less than 70% among in-center HD patients, based on results from matched observational studies.

**Methods:**

This simulation study adopted the perspective of the German statutory health insurance. A Markov (cohort) state-transition model was used to project the effects of the ACM over the remaining lifetime of patients. All costs were expressed in 2024 euros, and both costs and quality-adjusted life years (QALYs) were discounted at a rate of 3% per year. To test the sensitivity of the results, one-way sensitivity analyses and a probabilistic sensitivity analysis were performed.

**Results:**

This study finds that ACM provides more QALYs and incurs lower costs compared to standard of care. The net monetary value of ACM is €38,423 per patient in the base case scenario. In the sensitivity analysis, the annual cost of erythropoiesis-stimulating agents emerged as the variable with the largest impact on the value of ACM. The probabilistic sensitivity analysis shows that 100% of cost-effect pairs fall within the dominant southeast quadrant, indicating cost-effectiveness.

**Conclusions:**

This modelling study demonstrates that ACM is cost-effective for managing anemia in German in-center HD patients.

## Introduction

Artificial intelligence (AI) applications in healthcare are widely regarded as having the potential to reduce healthcare spending [1]. However, many cost projections are limited by a lack of empirical evidence on the costs and benefits of AI applications. One clinical field that has been underserved by AI to date is nephrology, despite the presence of large datasets and high unmet need [2]. An example application is the prediction of end-stage kidney disease (ESKD) in immunoglobulin A nephropathy [3].

Chronic kidney disease (CKD) is divided into five stages based on glomerular filtration rate [4]. Stage 5, referred to as ESKD, is typically treated with kidney replacement therapies such as dialysis or transplantation. An almost universal complication of CKD is anemia, which significantly reduces the quality of life (QoL) of patients and is associated with numerous symptoms and complications [5]. In CKD patients, anemia is typically managed with iron replacement and erythropoiesis-stimulating agents (ESAs). Patients receiving ESAs are maintained at a target hemoglobin (Hb) range well below the normal range, typically between 10 and 12 g/dL [6]. Administering ESA to maintain higher Hb levels increases the risk of death and serious cardiovascular reactions such as stroke, myocardial infarction, and heart failure [7]. Therefore, a rational and careful consideration of the risks and benefits is mandatory when correcting anemia in CKD patients [5].

The Anemia Control Model (ACM) is a certified medical device based on an AI core that supports nephrologists in making decisions related to anemia therapy. Specifically, ACM is designed for adult patients with ESKD who undergo in-center hemodialysis (HD) and suffer from secondary anemia [6]. This software application provides physicians with dosage recommendations for optimal ESA and iron therapy to maintain patient Hb and ferritin levels within the targeted range while minimizing the prescription of iron supplementation and ESAs. Physicians validate ACM suggestions and ultimately decide whether to implement ACM’s recommendations.

For patients on ESAs, ACM’s target Hb is centered around 11 g/dL. Following international guidelines [4, 8], ACM recommends interrupting ESA therapy when Hb reaches or exceeds 13 g/dL.

Observational studies have investigated the impact of ACM on patient time within the Hb target of 10 and 12 g/dL. A single-arm pre- and post-study conducted in three HD clinics across different European Union (EU) countries showed that ACM increases the percentage of Hb values within the target range by 20% [6]. However, this study was neither controlled nor blinded. Bucalo et al. [9] also conducted a single-arm study with an interrupted time-series design, where each patient served as their own control over time (blinding was not reported). This design helped limit selection bias and confounding by controlling for time-invariant patient characteristics. Conducted in two HD centers in Spain, this study similarly demonstrated an increase in Hb values within the target range by 14% upon reintroducing ACM, though the authors noted that the sample size and follow-up time were insufficient to assess the impact of ACM on cardiovascular morbidity and mortality.

Two newly conducted matched observational studies have evaluated the real-world effectiveness and safety of ACM in the Fresenius Medical Care NephroCare network. The first observational study involved a sample of 57,382 HD patients (age ≥ 18 years, kidney replacement therapy ≥ 90 days) treated in dialysis clinics owned by Fresenius Medical Care (FME) [10]. Data were collected from ten European countries (excluding Germany) between June 1, 2013, and December 31, 2019, prior to the COVID-19 pandemic. The total number of patient-months amounted to 1,525,960. The mix of participating clinics changed over time. For each ACM record, three corresponding matches were selected from non-ACM clinics classified according to the target achievement rate prior to the study index date. Non-ACM clinics were classified into three performance tiers: clinics performing at the international standard of care [11–13], defined by less than 70% of patients within the Hb target range prior to the study period (tier 1); clinics performing above the international standard of care (i.e., between 70% and 80% of patients within the Hb target range, tier 2); and clinics of excellence where more than 80% of patients achieved the therapeutic target (tier 3). A propensity score representing the likelihood of receiving a confirmed ACM ESA suggestion was used to inform the matching. The study demonstrated a significant impact of ACM on Hb target achievement (between 10 and 12 g/dL), inappropriate ESA administration (defined as ESA use in patients with Hb levels above 12 g/dL), and severe anemia (< 9 g/dL).

The second observational study [14] compared hospitalization and mortality rates between ACM users and non-users. Patients consistently treated according to ACM suggestions were matched on a 1:1 basis using a propensity score to a sample of patients who did not receive any ACM-based treatment. Overall, 3,900 patients were matched. During one year of follow-up, ACM users showed a 17% reduced risk of hospitalization (*p* < 0.01). In a sensitivity analysis (SA) based on propensity score covariate adjustment, which included approximately 3,800 ACM patients and 17,000 non-user controls, ACM users exhibited a 13.3% reduction in hospitalization risk compared to non-users (*p* < 0.01). Disease-specific hospitalization was not investigated due to potential misclassification (cardiovascular admissions) or unavailable data (anemia-specific hospitalizations). The impact on all-cause mortality was not significant (*p* = 0.5). It is important to note that inpatient mortality was captured by the endpoint of all-cause mortality.

Given the new evidence on the real-world effectiveness and safety of ACM, the cost-effectiveness of ACM remains to be investigated. Therefore, the purpose of this study is to analyze the cost-effectiveness of ACM compared to standard of care in Germany—defined as the absence of ACM and a Hb target achievement rate of less than 70% among in-center HD patients—from the perspective of the German statutory health insurance (SHI) system, based on the two newly conducted matched observational studies. To the best of our knowledge, this is the first cost-effectiveness analysis of an AI-based application in dialysis patients. The study also calculates the maximum price at which ACM would be acceptable to payers. As a country of application, we chose Germany, where 87,255 dialysis-dependent patients were insured by the SHI in 2017, with more than 90% of dialysis patients treated in-center [15]. By 2040, the number of dialysis-dependent patients is expected to increase by approximately 20–23%, reaching around 120,000 to 123,000 individuals [15].

## Methods

### Cost-effectiveness

As stated above, the research question addressed by this study is whether connecting a dialysis clinic to ACM is cost-effective—particularly for clinics performing at the international standard of care, defined as having less than 70% of patients achieving the Hb target range. In supplementary analyses, ACM was also compared against clinics performing above the international standard of care (i.e., between 70% and 80% of patients within the Hb target range) and clinics of excellence where more than 80% of patients achieved the therapeutic target. The research question is of primary interest to payers. Therefore, the economic evaluation is conducted from the perspective of the SHI, which covers approximately 73 million inhabitants in Germany [16]. Hence, the study accounts for direct medical and direct nonmedical costs. Direct medical costs included routine hemodialysis care, hospitalizations, potential savings from reduced ESA use, and costs associated with ESA-induced hypertension—specifically antihypertensive medication (atenolol) and outpatient visits for its management. Direct non-medical costs were captured within the total annual costs of dialysis patients, particularly through transportation expenses. From the SHI perspective, savings from reducing ESAs are relevant because they are reimbursed outside the weekly flat-rate fees for HD supplies and services [17].

A cost-utility analysis using quality-adjusted life-years (QALYs) as a measure of health benefits was conducted over the remaining lifetime of a patient. Based on the cost-utility analysis, we calculated the net monetary benefit (NMB) of ACM using the following equation [18]:

$$\:NMB=\lambda\:\cdot\:\varDelta\:E-\varDelta\:C,$$

where $\:\lambda\:$ is the maximum willingness-to-pay, $\:\varDelta\:C$ denotes incremental costs, and $\:\varDelta\:E$ represents incremental effects. The maximum willingness-to-pay was defined by the cost-utility ratio of treatment for HD patients (without ACM) in Germany. This ratio, calculated as part of the analysis, reflects the German SHI’s willingness to pay for treating patients with ESKD. The comparator of HD was assumed to be no (dialysis) treatment resulting in immediate death. The NMB was annualized by dividing the total NMB by the remaining life expectancy.

For the base-case analysis, both costs and effects were discounted at an annual rate of 3% [19]. In the SA, we employed rates of 0% and 5% [19]. The base case assumed continuous use of ACM throughout a patient’s remaining lifetime with a constant effect. In a SA, we conservatively assumed that ACM is used only for one year. The SA also considered patients with a relatively short HD treatment period due to transitions from in-center HD to other kidney replacement therapies.

This study was reported in accordance with the Consolidated Health Economic Evaluation Reporting Standards (CHEERS) 2022 checklist [20]. A completed checklist is provided in Appendix 1.

### Model

**Fig. 1:**
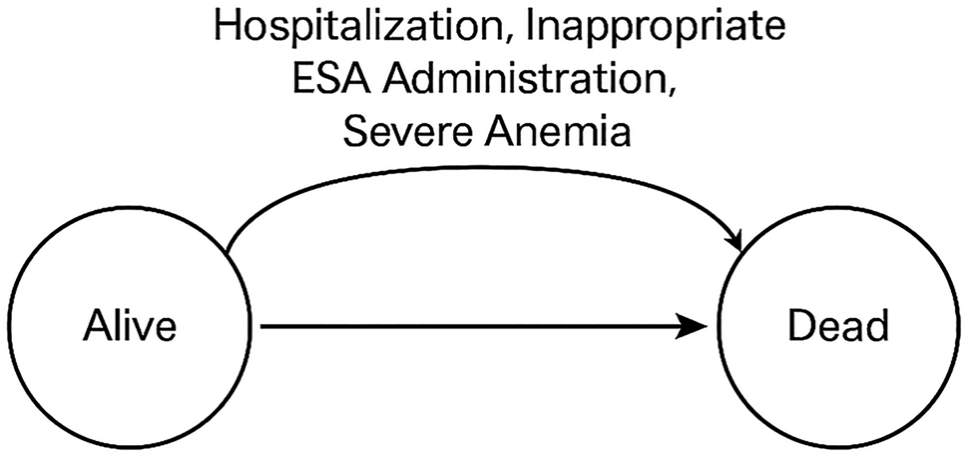
Markov model structure for evaluating the cost-effectiveness of the Anemia Control Model in in-center hemodialysis patients. Note: ESA: erythropoiesis-stimulating agent

This simulation study constructed a Markov (cohort) state-transition model to simulate the clinical and economic outcomes of 1,000 in-center HD patients with or without ACM. Our Markov model contains two health states - alive and dead. All patients entered the model within the alive state. Hospitalizations, inappropriate ESA administration, and severe anemia were modelled within the alive state (Fig. 1). Importantly, these endpoints were not assumed to persist over an entire annual cycle but were incorporated as monthly event probabilities based on observed incidence rates.

We did not include Hb target achievement as a separate endpoint due to considerable overlap with the definition of severe anemia and better alignment of disutilities from low Hb levels with the definition of severe anemia (see Results). The baseline characteristics at entry into the simulation were defined by the variables used for adjusting survival probabilities in the ERA-EDTA Registry [21]: age, 67 years; gender, 63% men; primary renal disease, 24% diabetes mellitus, 19% hypertension/renal vascular disease, 11% glomerulonephritis, and 46% other causes. In the SA, we considered patients who had been on in-center HD for 5 years already.

To define transitions to the death state, we applied the mortality rates for dialysis patients as determined above unless age-specific mortality in the general population was higher. For this purpose, we used the mortality table from the German Federal Office of Statistics [22].

Patients may transit to death at any time; however, they do not switch to the other treatment arm. During each cycle, patients accumulate QALYs and costs. A 12-month cycle length was chosen to align with the annualized reporting of key outcomes in the underlying observational studies [14], including hospitalization and survival. While inappropriate ESA use and severe anemia were reported as monthly averages [10], these metrics did not vary over time, which limits the value of modeling at a finer temporal resolution. Consequently, a shorter cycle length (e.g., monthly or quarterly) would introduce complexity without improving the accuracy of the estimates, as the model would rely on uniform values across cycles. From a health economic perspective, using annual cycles is also consistent with budgeting and decision-making horizons of payers such as the German SHI.

The life-table method of estimating state membership [23] was applied to both costs and life-years based on the assumption that transition events occur, on average, halfway through each 12-month cycle. The simulation was conducted up to the age of 100 years, thereby establishing a time horizon of 33 years in the base case. The cutoff was chosen because no official German mortality data are available beyond this age. Furthermore, at the age of 100, the proportion of patients still alive is less than 0.1% in both arms.

All cost-effectiveness calculations were conducted using Microsoft Excel (Redmond, WA, USA).

### Mortality

All input data are presented in Table 1. Given the absence of recent data on the annual mortality of the German HD population before the COVID-19 pandemic, we used survival data from the ERA-EDTA Registry [21], covering renal centers of 28 EU countries, including Germany. We applied age, sex, and type of kidney disease-adjusted survival data to represent the EU28 dialysis population. The German Institute for Quality Assurance and Transparency in Health Care [32] also referenced data from the ERA-EDTA Registry [21]. Survival probabilities between two and five years after dialysis initiation were approximated using geometric interpolation. Specifically, we assumed a constant annual hazard rate between years 2 and 5 and derived the annual survival probability accordingly. This approach preserves the internal consistency of the registry-reported endpoints while providing a transparent method to estimate annual survival in the absence of more granular longitudinal data. Beyond five years, we assumed a constant mortality rate using an exponential survival curve, also assumed in the United States Renal Data System annual report [33]. Given the uncertainty of applying this assumption to the German population—due to differences in healthcare systems, patient demographics, and treatment protocols—we varied the annual mortality rate within a plausible range (10–20%) in a sensitivity analysis.

**Table 1:** Input values and distributions used in the base case and sensitivity analysis. All costs are in Euros

Input	Mean (range)	Reference
**Clinical data**		
Survival probability after dialysis initiation:		Table A.5.1 in [21]
Year 1	0.857 (95% CI: 0.856–0.859)	
Year 2	0.755 (95% CI: 0.752–0.757)	
Year 5	0.474 (95% CI: 0.471–0.477)	
Hospitalizations per year (no ACM)	0.836 (0.775–0.836)	[14]
ACM vs. no ACM, ARR:		
Hospitalizations	0.126 (95% CI: 0.063–0.189)	[14]
ACM vs. standard of care (tier 1):		
Inappropriate ESA administration (RR)	0.37 (95% CI: 0.35–0.38)	[10]
Difference in ESA-free months	0.186 (95% CI: 0.1818–0.1902)	[14]
Severe anemia (RR)	0.41 (95% CI: 0.39–0.44)	[10]
ACM vs. above international standard of care (tier 2):		
Inappropriate ESA administration (RR)	0.49 (95% CI: 0.47–0.51)	[10]
Difference in ESA-free months	0.129 (95% CI: 0.1244–0.1336)	[14]
Severe anemia (RR)	0.64 (95% CI: 0.61–0.68)	[10]
ACM vs. clinics of excellence (tier 3):		
Inappropriate ESA administration (RR)	0.66 (95% CI: 0.63–0.69)	[10]
Difference in ESA-free months	0.097 (95% CI: 0.0928–0.1012)	[14]
Severe anemia (RR)	0.94 (95% CI: 0.88–1.00)	[10]
Standard of care (tier 1)		
Inappropriate ESA administration, events per patient month	0.1079 (95% CI: 0.1059–0.11)	[10]
Severe anemia, events per patient month	0.0490 (95% CI: 0.0477–0.0505)	[10]
Above international standard of care (tier 2)		
Inappropriate ESA administration, events per patient month	0.0818 (95% CI: 0.08–0.0837)	[10]
Severe anemia, events per patient month	0.0316 (95% CI: 0.0305–0.0328)	[10]
Clinics of excellence (tier 3)		
Inappropriate ESA administration, events per patient month	0.0604 (95% CI: 0.0588–0.0620)	[10]
Severe anemia, events per patient month	0.0217 (95% CI: 0.0207–0.0227)	[10]
Hypertension in ESA vs. control arm, OR	2.10 (95% CI: 1.22–3.59)	[24]
Probability of hypertension in control arm	0.92	[25]
**Economic data**		
Preference weight of HD treatment	0.69 (95% CI: 0.59–0.80)	[26]
Disutility of severe anemia	0.03 (0–0.03)	[27]
Annual cost of an HD patient	54,604 (95% CI: 53,514 − 55,693)	[15]
Annual cost of ESAs	5,200 (1,529–8,918)	[28]
Reimbursement per hospitalization based on DRG payment	4,210.59	[29]
Cost of atenolol (100 tablets, 100 mg)	23.31	[30]
Cost of outpatient visit related to hypertension	18.34	[31]
Annual discount rate, %	3 (0–5)	[19]

ACM: Anemia Control Model; CI: confidence interval; DRG: Diagnosis-Related Group; HD: hemodialysis; OR: odds ratio; RR: relative risk; ESA: erythropoiesis-stimulating agent

An evidence synthesis of the two observational studies [10, 14] was not conducted, as each study produced distinct clinical parameters for the model. Appendix 2 provides a critical appraisal of both studies using the STROBE checklist [34].

### Costs

The costs of ACM refer to the delivery and implementation of the intervention, which is an add-on to the current standard of care. These costs are fixed and do not depend on the number of clinics, meaning that adding another clinic incurs no additional costs unless a different clinical information system is used. From a SHI perspective, the delivery and implementation costs of ACM are zero as ACM is not reimbursed.

To estimate the annual cost of a hemodialysis (HD) patient, we used a published SHI-based estimate of €54,604 (in 2017 euros) based on statutory health insurance claims data, which includes transportation costs (€6,929), hospitalizations, pharmaceuticals, and non-dialysis-related healthcare costs [15]. This aligns with another study reporting costs of €44,374 in 2014, excluding transportation but including non-dialysis-related costs [35].

### Tariffs

Hospitalization costs were based on the 2025 national base rate of the German Diagnosis-Related Group (DRG) catalogue [29]. By multiplying the cost of a hospitalization by the probability of avoiding one, we calculated savings from ACM.

To estimate savings from reduced inappropriate ESA administration, we used actual GKV net costs for ESAs after applying legally mandated discounts [28]. In addition, we considered savings from the increased probability of not administering ESA. Although including this on top of the reduction in inappropriate ESA administration introduces a potential risk of double counting, the overlap is likely small, as the reduction in inappropriate use accounts for only a minor portion of the overall reduction in ESA consumption [10]. To address this uncertainty, only savings from the increase in the probability of not administering ESA were considered in a sensitivity analysis.

Furthermore, we accounted for a reduction in adverse events (AEs) due to less frequent ESA prescriptions. A recent Cochrane review [24] on anemia management in adults with CKD found that ESAs significantly reduce the need for blood transfusions but also increase the odds of developing hypertension. However, from the SHI perspective, avoiding blood transfusions does not result in cost savings, as transfusions are already covered under the DRG flat-rate payment. Therefore, our analysis focused only on the cost implications of increased hypertension.

To estimate the expected increase in hypertension incidence attributable to ESA use in dialysis patients, we used a baseline hypertension prevalence of 92%, based on data from ESKD patients in Vienna, Austria [25], which is considered relevant given the similarities between German-speaking healthcare systems. We applied the odds ratio (OR) for a commonly prescribed ESA in Germany (epoetin alfa), as reported in the Cochrane review, by converting the baseline prevalence to odds, applying the OR to estimate new odds under ESA treatment, and converting the result back to prevalence. This approach yielded an estimated 4% increase in hypertension prevalence.

We assumed atenolol (100 mg three times weekly post-dialysis) as the most frequently used first-line antihypertensive in dialysis patients in Germany, particularly for those treated with ESAs, due to its cardiovascular benefits, dialyzability, and minimal interaction with ESAs. For atenolol, we used net costs after legally mandated SHI discounts. Additionally, we assumed two extra outpatient visits per year due to hypertension, costed according to code 03000 from the German outpatient reimbursement schedule [31].

Costs were adjusted to year 2024 euros based on the German consumer price index.

### Preference-based quality of life

In the two observational studies, QoL data were not collected for patients with and without ACM. Therefore, the utility score for HD without ACM was based on a systematic review and meta-analysis of studies published up to 2010 by Wyld et al. [26]. The review included all studies that either reported utilities directly or where utilities could be calculated from the Short Form (36) Health Survey (SF-36) or SF-12 health surveys using a peer-reviewed algorithm.

The impact of ACM on preference weights was modelled based on the relationship between Hb levels and preference weights in HD patients. To this end, we conducted a literature search in PubMed on April 22, 2024, using the following search algorithm: *(hemodialysis OR haemodialysis OR dialysis[Title/Abstract])) AND (“eq-5d” OR “utility“[Title/Abstract]) AND ((quality of life[Title]) AND (hemoglobin OR haemoglobin[Title/Abstract])*. This was followed by a hand search of bibliographies for relevant articles.

### Sensitivity analysis

Along with one-way SAs, we conducted a probabilistic sensitivity analysis (PSA) using Monte-Carlo simulation to assess how simultaneous changes in several variables affect the NMB of ACM. With 1000 samples, we obtained stable results. The PSA used the parameter ranges reported in Table 1. Probabilities and utilities were assumed to follow a beta distribution because they are restricted to values between 0 and 1. Relative risks and odds ratios were assumed to follow a log-normal distribution after logarithmic transformation to account for their positive values and skewed distribution. The costs of dialysis treatment were assumed to follow a gamma distribution to reflect their positive, right-skewed nature. The annual cost of ESA use was assumed to follow a triangular distribution, based on available minimum and maximum values, with the most likely value approximated as the midpoint between them. The absolute risk reduction of hospitalizations and the disutility of severe anemia were assumed to follow a uniform distribution due to the lack of detailed distribution information (i.e., confidence intervals). The difference in ESA-free months between ACM and comparator arms was modelled using a normal distribution, justified by the large sample sizes and narrow, symmetric confidence intervals. To reflect the clinical interdependence of model parameters, we incorporated correlation into the PSA. Specifically, we assumed a positive correlation between the absolute risk reduction in hospitalizations and the relative risk of severe anemia, as both outcomes are influenced by anemia control and ESA management. In the absence of empirical correlation estimates, we modelled this relationship using a Cholesky decomposition approach with a correlation coefficient of 0.5.

## Results

### Literature search

The PubMed search on the relationship between Hb levels and preference weights in dialysis patients yielded eight results. An additional study was identified through a hand search. We excluded studies using Hb threshold values that did not match the definitions of the Hb target range or severe anemia used in our study. We found one study that reported a disutility 0.03 for dialysis patients with an Hb level below 10 g/dL (Fig. 1a in [27]). The study was based on the three-level version of the EuroQol-5D-3 L™ (EQ-5D™) questionnaire and conducted as a multinational survey that included Germany. According to personal communication with the corresponding author, country-specific tariffs were used to convert EQ-5D-3 L responses into utility scores for each participating country, except for China, where the UK tariff was applied. Given that the study did not report utilities separately for patients with or without ESA therapy, the preference weight of Hb levels above the target range (i.e., 12 g/dL) was not considered. Thus, we focused only on severe anemia as an endpoint, applying the disutility of 0.03 to patients with severe anemia, which is conservative given that patients with an Hb below 8 g/dL were reported in the same study to have a disutility of 0.04 [27]. However, the authors caution that the observational and cross-sectional nature of this survey limits the ability to assess causality between exposures and outcomes. Therefore, the results from this survey can only be interpreted as associative. Consequently, we assumed no effect on preference-based QoL in a SA.

### Base case

As shown in Table 2, the cost-utility ratio of treating HD patients without ACM compared to no (dialysis) treatment is €100,858 per QALY gained. This ratio reflects the willingness of the German SHI to pay for treating patients with ESKD. ACM dominates no ACM by reducing the incidence of hospitalizations, ESA utilization, and associated adverse events, while increasing QALYs through improved QoL. Based on a QALY gain of 0.06, the total NMB of ACM is €38,423 per patient in the base case, which translates to €6,726 per patient per year.

**Table 2:** Costs, effects, and cost-effectiveness of the Anemia control model (ACM) and no ACM. All costs are in Euros

Discounting	Treatment	Lifetime costs	Life years	QALYs	Incremental costs per life year gained	Incremental costs per QALY gained
3%	ACM	353,551	5.71	3.89	Dominates	Dominates
	No ACM	386,022	5.71	3.83	67,575	100,858
No discounting	ACM	435,311	7.03	4.79	Dominates	Dominates
	No ACM	475,290	7.03	4.71	67,575	100,858

QALY: quality-adjusted life year

### Comparator analysis

Comparing ACM against clinics performing above the international standard of care (i.e., between 70% and 80% of patients within the Hb target range) and clinics of excellence where more than 80% of patients achieved the therapeutic target yields total NMBs of €23,761 and €13,206 per patient in the base case, translating to €4,159 and €2,312 per patient per year, respectively.

### Sensitivity analysis

**Fig. 2:**
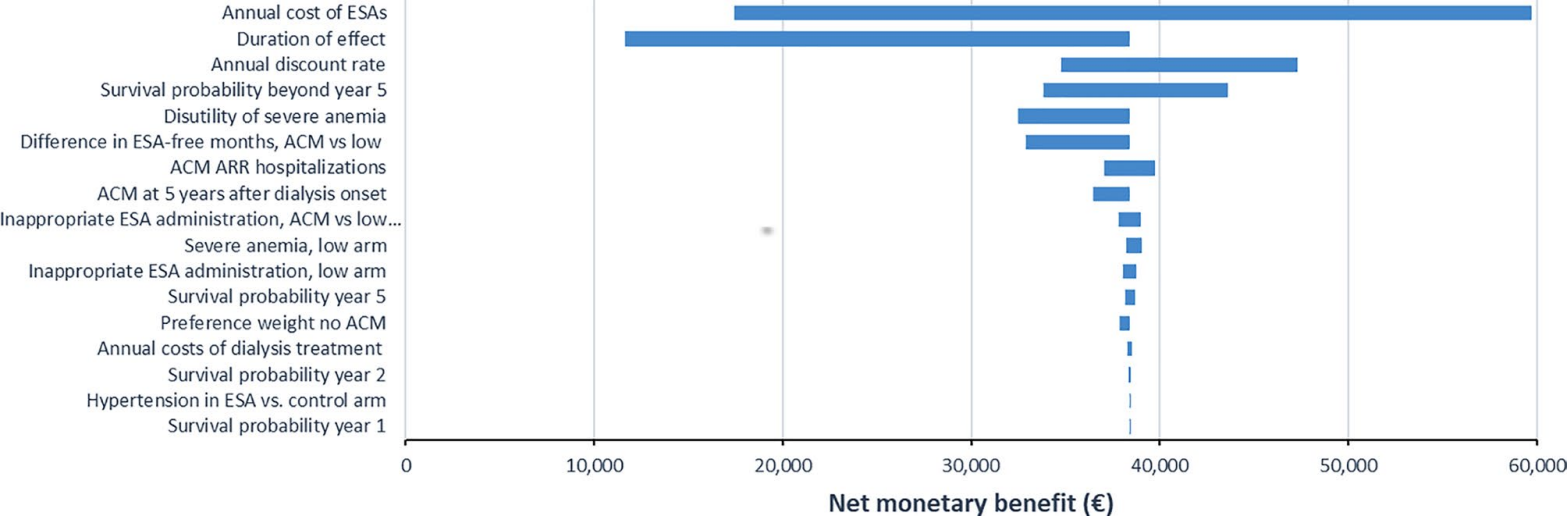
Tornado diagram demonstrating the results of the one-way sensitivity analysis. Notes: Variables are ordered by impact on the net monetary benefit of the Anemia Control Model (ACM). ESA: erythropoiesis-stimulating agent; ARR: absolute risk reduction

**Fig. 3:**
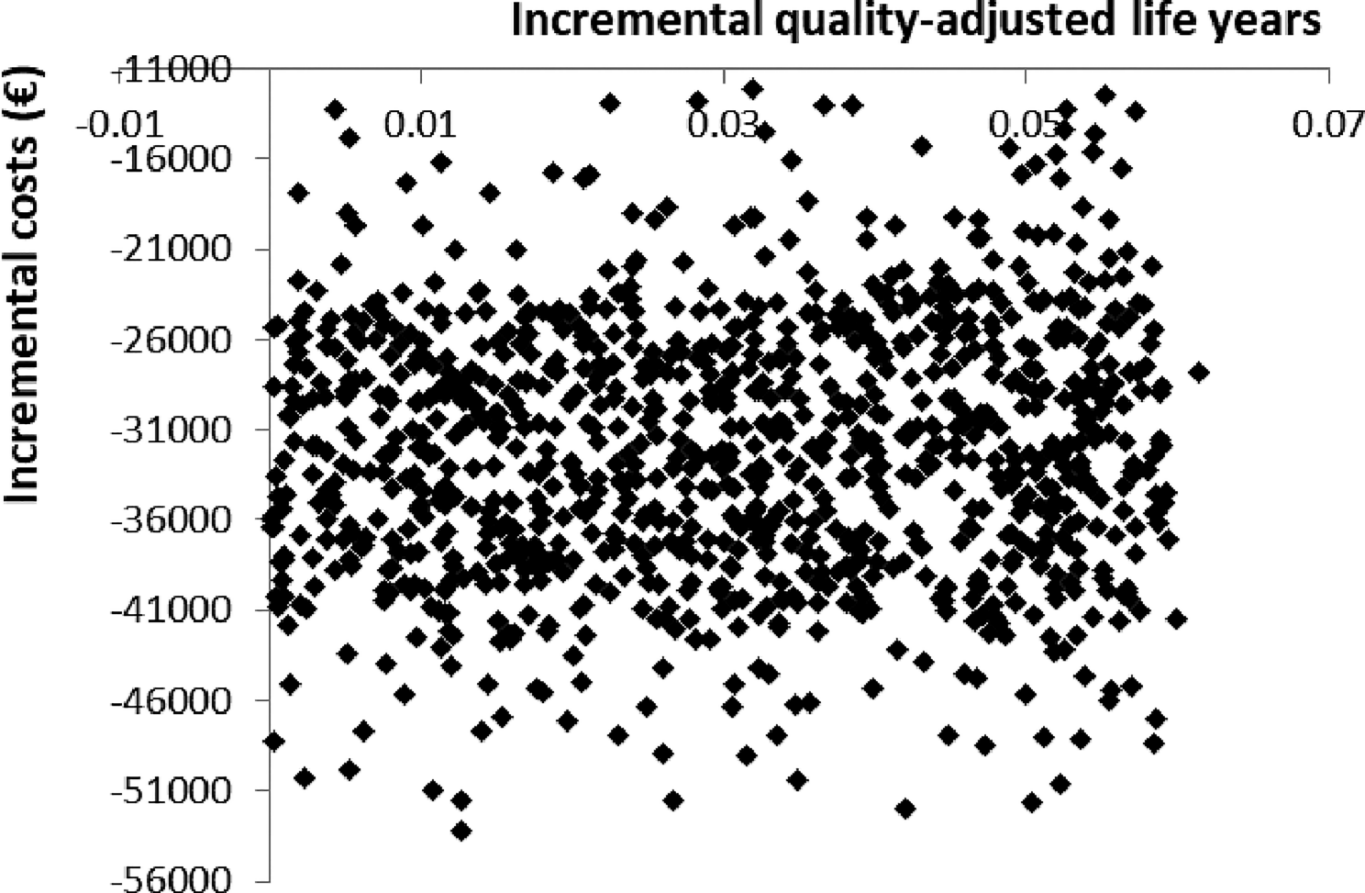
Scatter plot of results from the Monte Carlo simulation

As shown in Fig. 2, the one-way SA confirmed a positive NMB under all model and input assumptions. The variable with the largest impact on the NMB of ACM is the annual cost of ESAs. The Monte Carlo simulation demonstrates that 100% of cost-effectiveness pairs fall in the dominant southeast quadrant (Fig. 3).

## Discussion

This study demonstrates that ACM dominates its comparator from the perspective of the German statutory health insurance by yielding more QALYs while reducing costs. The cost-effectiveness of ACM is a logical outcome, primarily because ACM is not reimbursed by the SHI, implying that the intervention costs are zero. Simultaneously, ACM is not associated with AEs or significant survival benefits. Arguably, even the inclusion of life extension costs, including those unrelated to dialysis treatment, would not alter this conclusion if survival gains were reimbursed at the current willingness-to-pay threshold for dialysis treatment. Given this rationale and assuming that the results from implementing ACM in the ten European countries are transferable, dominance of ACM from a payer perspective is likely in other jurisdictions as well.

The net monetary value created by ACM per patient indicates the maximum willingness-to-pay for ACM per patient covered by the SHI. Total and annual values yielded by ACM are particularly pertinent, considering that the projected significant increase in the number of patients requiring dialysis treatment in Germany due to demographic changes alone [15]. The annual value generated by ACM could serve as a reference point for a fixed subscription fee that sickness funds may be willing to pay the ACM provider annually. This approach is consistent with the principle of value-based pricing, whereby the cost of implementing ACM—borne by dialysis clinics—is not directly relevant to the sickness funds.

The clinical improvements in Hb target achievement and reduced ESA consumption observed in the underlying real-world study varied by the performance level of comparator clinics. The greatest net monetary value was observed compared to patients treated in clinics performing at the level of international standards of Hb target achievement, with gradually less evident net monetary value for clinics performing above the international benchmark.

While the EU mortality data on dialysis patients used in this study include patients on peritoneal dialysis [21], patient characteristic (63% men, average age of 67 years) seem representative of the German dialysis population. An analysis of all German SHI patients (*n* = 46,039), who have initiated dialysis treatment between 2013 and 2017, including patients on peritoneal dialysis, showed that 62% were male and the average age was 68 years [36]. However, there may be minor differences in the distribution of primary renal disease, particularly in the share attributed to diabetes, which tends to be slightly higher in some German datasets. Despite these nuances, the ERA-EDTA dataset offers a reasonable approximation of the German dialysis population and is referenced by German health authorities (e.g., IQTIG [32]), thereby supporting its use for population-level modeling in this context.

In the underlying observational studies [10, 14], care settings were relatively standardized due to the shared, protocol-driven environment of clinics within the provider network. These clinics operated under harmonized clinical pathways, data documentation systems (EuCliD^®^), and anemia management protocols, which helped reduce variability in treatment delivery and patient monitoring across countries. As such, the use of a cohort-based Markov model appears sufficient to capture the system-level impact of ACM on key outcomes such as hospitalization rates, survival, and QALYs.

While a microsimulation model could theoretically allow for more granular modeling of individual patient trajectories—including intermediate health states such as living with complications (e.g., fistula thrombosis, ESA-related adverse events) or varying levels of anemia control—the underlying observational studies did not report outcomes at this level of clinical detail. Therefore, the use of a simpler Markov model was appropriate given the data structure and the primary objective of the study: to estimate average cost-utility from the perspective of the SHI.

In future evaluations, particularly those aimed at national rollouts across more heterogeneous care settings or targeting subpopulations, the adoption of a microsimulation framework could be considered. For the current analysis, however, the Markov model offers a transparent, robust, and fit-for-purpose structure aligned with the granularity of the available evidence.

There are several reasons why the study may underestimate the value of ACM. First, it does not account for potential follow-up costs after hospital discharge, hence underestimates savings from avoided hospitalizations. Second, it does not consider other potentially relevant endpoints such as arteriovenous fistula thrombosis. Third, our study likely underestimates the utility gain from preventing ESA overuse because it does not model disutility from AEs secondary to ESA therapy. Fourth, the comparator for HD was assumed to be no (dialysis) treatment resulting in immediate death, which likely underestimates the willingness-to-pay threshold. A more realistic comparator, such as conservative management, would likely increase the cost-utility ratio of HD, thereby increasing the value of ACM [37].

Adopting a societal perspective, which includes not only broader impacts such as productivity losses and patient time costs but also the full costs of implementing ACM, may lead to different results compared to the SHI perspective. While from the perspective of static efficiency ACM costs are considered sunk, from a dynamic societal perspective, which incentivizes innovation (i.e., ACM), they remain relevant. That is, fully accounting for ACM costs avoids overly generous incentives for innovation. However, a societal perspective might reinforce the results of this study by accounting for potential savings from reduced co-payments for hospitalizations, decreased time spent seeking medical care, less transportation costs to and from medical facilities, and reduced productivity loss. Moreover, a societal perspective would consider potential reductions in physician time, resulting from fewer data checks (e.g., Hb levels) necessary in ACM clinics.

In the two observational studies underlying the clinical parameters used in the cost-effectiveness model [10, 14], the risk of indication bias was minimized, as ACM activation was determined first at the country level and subsequently at the clinic level by the medical director. Despite this quasi-experimental design and the use of propensity score matching to further reduce indication bias related to the acceptance of ACM suggestions, we cannot fully rule out the possibility of selection or confounding bias, as it cannot be excluded with certainty that clinic-level policy decisions regarding ACM acceptance were influenced by patients’ clinical characteristics.

The generalizability of the results from the matched cohort studies may be limited, as neither study included data from German dialysis centers. While both studies were conducted in European clinics operating under similar quality improvement protocols, differences in healthcare infrastructure, treatment practices, and regulatory environments may impact the applicability of the findings to the German context. Nevertheless, the comparable clinical processes and centralized anemia management protocols used across countries within the same provider network support a cautious extrapolation to Germany.

More broadly, the fact that data were collected from ten European countries with diverse healthcare financing schemes—including both Bismarckian and Beveridge-type systems—enhances the potential transferability of findings to other international settings. The standardized implementation of ACM across different countries within the provider network, along with consistent improvements in anemia management performance, suggests that the clinical effectiveness of ACM may be generalizable to other advanced healthcare systems with comparable levels of digital infrastructure and physician oversight.

However, the economic model used in this study reflects the perspective of the German SHI system, which may limit its direct applicability to countries with different reimbursement mechanisms. For example, in Beveridge-type systems—where healthcare is primarily tax-funded and provider reimbursement structures vary significantly—the cost-saving implications of reducing ESA use or hospitalizations may differ. As such, while clinical outcomes may translate more broadly, economic results should be interpreted with caution when applied to other healthcare financing contexts.

Furthermore, differences in physician autonomy, data infrastructure, and incentives for technology adoption across systems may affect the implementation and effectiveness of AI-driven decision support tools like ACM. Therefore, while the clinical benefits observed under standardized protocols may be broadly relevant, the financial and organizational implications of ACM require contextual adaptation to the specific features of each healthcare financing and delivery system.

The ACM is a CE-marked medical device designed as a clinical decision support tool, not as a replacement for physician judgment. While it relies on AI-based algorithms, final treatment decisions remain with the nephrologist, ensuring human oversight and accountability. This mitigates ethical concerns regarding autonomy and patient safety. Nonetheless, ethical implications must be continuously monitored, particularly regarding algorithmic transparency, data privacy, and potential biases in AI training data. As ACM is implemented “as is” within a standardized provider network, the current findings may not fully reflect the implications of deploying ACM in more heterogeneous, less-regulated environments.

Beyond ethical considerations, practical implementation challenges may also limit the scalability of AI-based tools like ACM. These include potential resistance from physicians due to concerns about loss of autonomy or trust in algorithmic recommendations, as well as technical barriers such as the need for seamless integration with existing electronic health record systems. Future research should explore the impact of ACM in settings with varying levels of digital infrastructure, regulatory oversight, and clinician engagement to support responsible and equitable adoption.

For future research, we also recommend investigating the impact of ACM on health-related QoL based on primary data, not only in patients with severe anemia but across the broader population of dialysis patients affected by ACM. Additionally, based on the result of the SA, we recommend assessing the effects of ACM over a longer time horizon in a prospective study.

As the present study adopts an SHI perspective, it does not explicitly model the resource implications for dialysis clinics where ACM is implemented. From a provider perspective, ACM may be associated with both additional costs (e.g., software integration, training) and cost savings (e.g., reduced ESA use, fewer complications). As such, the net value generated for the payer—used here to inform a potential subscription fee—may not fully reflect the economic impact at the provider level. Future research should consider alternative modeling frameworks, such as clinic-based budget impact models, to more comprehensively assess the distribution of costs and benefits across stakeholders. This would support more informed value-based pricing and contracting strategies that better align incentives across the healthcare system.

## Appendix 1. Completed CHEERS 2022 checklist for reporting of health economic evaluations

**Table:** 

CHEERS item	Description	Reported in manuscript
1. Title	Identifies study as an economic evaluation and specifies interventions	Title
2. Abstract	Structured summary with context, methods, results, and alternative analyses	Abstract section
3. Background/Objectives	Study context, question, and policy/practice relevance	Introduction
4. Health economic analysis plan	Not explicitly mentioned	*Not reported*
5. Study population	Age, gender, disease characteristics described	Methods – Model
6. Setting and location	Germany, SHI perspective; observational data from European NephroCare clinics	Introduction, Methods
7. Comparators	ACM vs. standard care (tier 1) and other performance tiers	Methods – Cost-effectiveness
8. Perspective	SHI perspective, explained and justified	Methods – Cost-effectiveness
9. Time horizon	Lifetime (up to age 100), justified	Methods – Model
10. Discount rate	3%, varied in SA to 0% and 5%, with rationale	Methods – Cost-effectiveness
11. Selection of outcomes	QALYs, hospitalization, inappropriate ESA use, anemia status	Methods – Cost-effectiveness, Model
12. Measurement of outcomes	From observational studies and literature	Methods – Mortality
13. Valuation of outcomes	Utility values from published literature and PubMed search	Methods – Preference-based QoL
14. Measurement and valuation of costs	Based on German SHI tariffs, DRGs, CPI index	Methods – Costs
15. Currency, price date, conversion	Euros, adjusted to 2024	Methods – Costs
16. Model rationale and description	Cohort-based Markov model, structure justified	Methods – Model
17. Analytics and assumptions	One-way and probabilistic SA, distributions reported	Methods – Sensitivity Analysis
18. Heterogeneity	Analyses across comparator tiers (tiers 1–3)	Methods – Cost-effectiveness
19. Distributional effects	Not addressed	Not reported
20. Uncertainty characterization	One-way and PSA, tornado diagram and Monte Carlo results	Results – Sensitivity Analysis
21. Stakeholder engagement	Not described	Not reported
22. Study parameters	Full list in Table 1 with ranges and references	Methods – Table 1
23. Main results summary	QALYs, cost, and ICERs reported; dominance shown	Results – Table 2
24. Effect of uncertainty	Discount rate and time horizon varied; PSA results described	Results – SA and Discussion
25. Effect of engagement	Not reported	Not reported
26. Study findings and limitations	Addressed extensively including ethics, generalizability	Discussion
27. Source of funding	Fresenius Medical Care Deutschland GmbH	Funding
28. Conflicts of interest	Declared in Declarations section	Declarations

## Appendix 2. Critical appraisal of observational studies using the STROBE checklist [34]

1. Garbelli et al.

Title: Usage of the Anemia Control Model Is Associated with Reduced Hospitalization Risk in Hemodialysis Journal: Biomedicines 2024, 12, 2219 Pages Referenced: 1–16.

**Table:** 

STROBE Item	Assessment	Page(s)
Title/Abstract	Design clearly mentioned; results summarized	1
Background/Rationale	Comprehensive clinical rationale and problem statement	2–3
Objectives	Clearly stated	3
Study Design	Retrospective matched cohort; clearly defined	3–4
Setting	Multi-country Fresenius NephroCare centers in Europe (excl. Germany)	3
Participants	Eligibility, matching, and exclusion criteria specified	3–4
Variables	Detailed definition of exposures, outcomes, and covariates	3–4
Data Sources/Measurement	Extracted from NephroCare EHRs; lab and demographic data sources listed	3
Bias	Propensity score matching, clinic-level activation policy	3–4
Study Size	*N* = 20,209 before matching, *N* = 1952 per group after matching	5–6
Quantitative Variables	Described for all major lab and clinical variables	6–7
Statistical Methods	Matching, regression models, and sensitivity analysis described	4, 7
Results– Participants	Flowchart included, exclusions and matching process explained	6
Results– Descriptive Data	Comprehensive tables before and after matching	6–9
Results– Outcome Data	Event rates, confidence intervals, incidence rate ratios	10
Main Results	Adjusted and unadjusted results for hospitalization and mortality	10
Other Analyses	Kt/V sensitivity analysis; unmatched patients analyzed	11
Key Results	Clear summary with link to objectives	11
Limitations	Residual confounding, OL-HDF confounding discussed	11–12
Interpretation	Cautious interpretation with reference to related work	11–12
Generalizability	Limited to European NephroCare network; Germany not included	12
Funding and Conflicts	Full author disclosures and funding statement provided	13–14

2. Garbelli et al.

Title: The Use of Anemia Control Model Is Associated with Improved Hemoglobin Target Achievement, Lower Rates of Inappropriate Erythropoietin Stimulating Agents, and Severe Anemia among Dialysis Patients Journal: Blood Purif. 2024;53(5) Pages Referenced: 1–13.

**Table:** 

STROBE Item	Assessment	Page(s)
Title/Abstract	Design, outcomes, and methods clearly stated	1
Background/Rationale	Detailed rationale on CKD anemia challenges	2
Objectives	Effectiveness and safety evaluation	2
Study Design	Multicenter, retrospective matched cohort	2–3
Setting	10 European countries, excluding Germany	3
Participants	Extensive eligibility and matching criteria	3–4, Fig. 1
Variables	Hb, ESA, iron use, anemia status	4–5
Data Source/Measurement	EuCliD^®^ database	3
Bias	Policy-level ACM activation, PS matching	4–5
Study Size	85,512 patient-months per group	6
Statistical Methods	Propensity score, regression, sensitivity analysis	4
Results	Detailed, stratified by tiers, with CIs	5–7
Limitations	Discussed confounding and PS limitations	11
Interpretation	Real-world and robust, contextualized with prior trials	10–11
Generalizability	Wide within NephroCare; not German-specific	11
Funding	No external funding; disclosed conflicts	11

## Data Availability

No datasets were generated or analysed during the current study.

## References

[1] Kalis B, Collier M, Fu R. 10 Promising AI Applications in Health Care. May 10. 2018. https://hbr.org/2018/05/10-promising-ai-applications-in-health-care

[2] Chaudhuri S, Long A, Zhang H, Monaghan C, Larkin JW, Kotanko P, Kalaskar S, Kooman JP, van der Sande FM, Maddux FW, Usvyat LA. Artificial intelligence enabled applications in kidney disease. Semin Dial. 2021;34(1):5–16. 32924202 10.1111/sdi.12915 PMC7891588

[3] Liu Y, Zhang Y, Liu D, Tan X, Tang X, Zhang F, Xia M, Chen G, He L, Zhou L, Zhu X, Liu H. Prediction of ESRD in IgA nephropathy patients from an Asian cohort: a random forest model. Kidney Blood Press Res. 2018;43(6):1852–64. 30537719 10.1159/000495818

[4] KDIGO CKD Work Group. KDIGO 2012 clinical practice guideline for the evaluation and management of chronic kidney disease. Kidney Int Suppl. 2013;3:1–150. 10.1038/ki.2013.243 23989362

[5] Macdougall IC, Eckardt KU. Anemia in chronic kidney disease. In: Johnson RJ, Feehally J, Floege J, editors. Comprehensive Clinical Nephrology e-Book. Elsevier Health Sciences; 2014.

[6] Barbieri C, Molina M, Ponce P, Tothova M, Cattinelli I, Ion Titapiccolo J, Mari F, Amato C, Leipold F, Wehmeyer W, Stuard S, Stopper A, Canaud B. An international observational study suggests that artificial intelligence for clinical decision support optimizes anemia management in hemodialysis patients. Kidney Int. 2016;90(2):422–9. 27262365 10.1016/j.kint.2016.03.036

[7] Manns BJ, Tonelli M. The new FDA labeling for ESA–implications for patients and providers. Clin J Am Soc Nephrol. 2012;7(2):348–53. 22266575 10.2215/CJN.09960911 PMC3280029

[8] Renal Association. Clinical practice guideline on anaemia of chronic kidney disease. Updated February 2020. https://ukkidney.org/sites/renal.org/files/Updated-130220-Anaemia-of-Chronic-Kidney-Disease-1-1.pdf

[9] Bucalo ML, Barbieri C, Roca S, Ion Titapiccolo J, Ros Romero MS, Ramos R, Albaladejo M, Manzano D, Mari F, Molina M. The anaemia control model: does it help nephrologists in therapeutic decision-making in the management of anaemia? Nefrologia (Engl Ed). 2018 Sep-Oct;38(5):491–502. 10.1016/j.nefro.2018.03.004 29875061

[10] Garbelli M, Bellocchio F, Baro Salvador ME, Chermisi M, Rincon Bello A, Godoy IB, Perez SO, Shkolenko K, Perez AS, Toro DS, Apel C, Petrovic J, Stuard S, Barbieri C, Mari F, Neri L. The use of anemia control model is associated with improved hemoglobin target achievement, lower rates of inappropriate erythropoietin stimulating agents, and severe anemia among dialysis patients. Blood Purif. 2024;53(5):405–17. 38382484 10.1159/000536181

[11] Zuo L, Wang M, Hou F, Yan Y, Chen N, Qian J, Wang M, Bieber B, Pisoni RL, Robinson BM, Anand S. Anemia management in the China Dialysis Outcomes and Practice Patterns Study. Blood Purif. 2016;42(1):33–43. 27045519 10.1159/000442741 PMC4919113

[12] Gaweda AE, Jacobs AA, Aronoff GR, Brier ME. Individualized anemia management in a dialysis facility - long-term utility as a single-center quality improvement experience. Clin Nephrol. 2018;90(4):276–85. 30049300 10.5414/CN109499 PMC6350237

[13] United States Renal Data System. 2017 Annual data report. https://www.usrds.org/media/2286/2017_volume_2_esrd_in_the_us.pdf

[14] Garbelli M, Baro Salvador ME, Rincon Bello A, Samaniego Toro D, Bellocchio F, Fumagalli L, Chermisi M, Apel C, Petrovic J, Kendzia D, et al. Usage of the Anemia control model is associated with reduced hospitalization risk in hemodialysis. Biomedicines. 2024;12(10):2219. 39457532 10.3390/biomedicines12102219 PMC11504963

[15] Häckl D, Kossack N, Schoenfelder T. Prävalenz, Kosten der Versorgung und Formen des dialysepflichtigen chronischen Nierenversagens in Deutschland: Vergleich der Dialyseversorgung innerhalb und außerhalb stationärer Pflegeeinrichtungen. Gesundheitswesen. 2021;83(10):818–28. 10.1055/a-1330-7152 PMC8497075 33450773

[16] Bundesgesundheitsministerium. Februar. Ergebnisse der GKV-Statistik KM1. Stand: Februar 2022. https://www.bundesgesundheitsministerium.de/fileadmin/Dateien/3_Downloads/Statistiken/GKV/Mitglieder_Versicherte/KM1_Januar_bis_Februar_2022_bf.pdf

[17] Kassenärztliche Bundesvereinigung. Einheitlicher Bewertungsmaßstab. Stand 2022/1.

[18] Stinnett AA, Mullahy J. Net health benefits: a new framework for the analysis of uncertainty in cost-effectiveness analysis. Med Decis Mak. 1998 Apr-Jun;18(2 Suppl):S68–80. 10.1177/0272989X98018002S09 9566468

[19] Institut für Qualität und Wirtschaftlichkeit im Gesundheitswesen. General methods. Version 7.0. Köln: IQWiG; 2023.

[20] Husereau D, Drummond M, Augustovski F, de Bekker-Grob E, Briggs AH, Carswell C, et al. Consolidated health economic evaluation reporting standards 2022 (CHEERS 2022) statement: updated reporting guidance for health economic evaluations. BMJ. 2022;376:e067975. 35017145 10.1136/bmj-2021-067975 PMC8749494

[21] Registry ERA-EDTA: ERA-EDTA Registry Annual Report 2019., Amsterdam UMC. location AMC, Department of Medical Informatics, Amsterdam, the Netherlands, 2021.

[22] Statistisches Bundesamt. Sterbetafeln 2018/2020. Wiesbaden: Statistisches Bundesamt; 2021.

[23] Barendregt JJ. The half-cycle correction: banish rather than explain it. Med Decis Mak. 2009;29(4):500–2. 10.1177/0272989X09340585 19571330

[24] Chung EY, Palmer SC, Saglimbene VM, Craig JC, Tonelli M, Strippoli GF. Erythropoiesis-stimulating agents for anaemia in adults with chronic kidney disease: a network meta-analysis. Cochrane Database Syst Rev. 2023;2(2):CD010590. 36791280 10.1002/14651858.CD010590.pub3 PMC9924302

[25] Königsbrügge O, Posch F, Antlanger M, Kovarik J, Klauser-Braun R, Kletzmayr J, Schmaldienst S, Auinger M, Zuntner G, Lorenz M, Grilz E, Stampfel G, Steiner S, Pabinger I, Säemann M, Ay C. Prevalence of atrial fibrillation and antithrombotic therapy in hemodialysis patients: cross-sectional results of the Vienna InVestigation of AtriaL Fibrillation and Thromboembolism in Patients on HemoDIalysis (VIVALDI). PLoS ONE. 2017;12(1):e0169400. 28052124 10.1371/journal.pone.0169400 PMC5213813

[26] Wyld M, Morton RL, Hayen A, Howard K, Webster AC. A systematic review and meta-analysis of utility-based quality of life in chronic kidney disease treatments. PLoS Med. 2012;9(9):e1001307. 22984353 10.1371/journal.pmed.1001307 PMC3439392

[27] van Haalen H, Jackson J, Spinowitz B, Milligan G, Moon R. Impact of chronic kidney disease and anemia on health-related quality of life and work productivity: analysis of multinational real-world data. BMC Nephrol. 2020;21(1):88. 32143582 10.1186/s12882-020-01746-4 PMC7060645

[28] Gemeinsamer Bundesausschuss. Vadadustat (symptomatische Anämie bei chronischer Nierenerkrankung (CKD)). Beschluss vom 22. November 2024. https://www.g-ba.de/downloads/91-1385-1091/2024-11-22_Geltende-Fassung_Vadadustat_D-1073.pdf

[29] AOK. Der errechnete Bundesbasisfallwert. https://www.aok.de/gp/verwaltung/landesbasisfallwerte/bundesbasisfallwert

[30] Gemeinsamer Bundesausschuss. Tragende Gründe zum Beschluss des Gemeinsamen Bundesausschusses über eine Änderung der Arzneimittel-Richtlinie: Anlage XII– Nutzenbewertung von Arzneimitteln mit neuen Wirkstoffen nach § 35a des Fünften Buches Sozialgesetzbuch (SGB V) Mavacamten (symptomatische hypertrophe obstruktive Kardiomyopathie (NYHA Klasse II–III)) Vom 1. Februar 2024. https://www.g-ba.de/downloads/40-268-10197/2024-02-01_AM-RL-XII_Mavacamten_D-962_TrG.pdf

[31] KBV, Kassenärztliche Bundesvereinigung. Einheitlicher Bewertungsmaßstab (EBM). Stand: 2. Quartal 2025. https://www.kbv.de/media/sp/EBM_Gesamt_-_Stand_2._Quartal_2025.pdf

[32] Institut für Qualitätssicherung und Transparenz im Gesundheitswesen. Beschreibung der Qualitätsindikatoren und Kennzahlen nach DeQS-RL. Nierenersatztherapie bei chronischem Nierenversagen einschließlich Pankreastransplantationen: Dialyse. Prospektive Rechenregeln für das Erfassungsjahr 2021. Berlin: IQTIG; 17.12.2020.

[33] United States Renal Data System. 2018 USRDS Annual Data Report. https://view.officeapps.live.com/op/view.aspx?src=https%3A%2F%2Fwww.usrds.org%2Fmedia%2F1787%2Fv2_c02_clincare_18_slides.pptxwdOrigin=BROWSELINK

[34] von Elm E, Altman DG, Egger M, Pocock SJ, Gøtzsche PC, Vandenbroucke JP, STROBE Initiative. The strengthening the reporting of observational studies in epidemiology (STROBE) statement: guidelines for reporting observational studies. Lancet. 2007;370(9596):1453–7. 18064739 10.1016/S0140-6736(07)61602-X

[35] Gandjour A, Armsen W, Wehmeyer W, Multmeier J, Tschulena U. Costs of patients with chronic kidney disease in Germany. PLoS ONE. 2020;15(4):e0231375. 32330140 10.1371/journal.pone.0231375 PMC7182232

[36] Zentralinstitut für die kassenärztliche Versorgung in der Bundesrepublik Deutschland. Routinedatenauswertung zu Hämo- versus Peritonealdialysen in Deutschland. 28.05.2019. https://www.zi.de/fileadmin/images/content/Projekte/MAUPD_Abschlussbericht_Zi_final.pdf

[37] Lévesque R, Marcelli D, Cardinal H, Caron ML, Grooteman MP, Bots ML, Blankestijn PJ, Nubé MJ, Grassmann A, Canaud B, Gandjour A. Cost-effectiveness analysis of high-efficiency hemodiafiltration versus low-flux hemodialysis based on the Canadian arm of the CONTRAST study. Appl Health Econ Health Policy. 2015;13(6):647–59. 26071951 10.1007/s40258-015-0179-0 PMC4661220

